# Validation and invariance testing of the English Short Physical activity enjoyment scale

**DOI:** 10.1371/journal.pone.0313626

**Published:** 2024-11-11

**Authors:** Susanne Weyland, Navin Kaushal, Julian Fritsch, Ulrich Strauch, Darko Jekauc

**Affiliations:** 1 Department of Health Education and Sports Psychology, Institute of Sports and Sports Science, Karlsruhe Institute of Technology, Karlsruhe, Germany; 2 Department of Health Sciences, School of Health & Human Sciences, Indiana University, Indianapolis, Indiana, United States of America; 3 Department of Sports Sciences, Sport and Exercise Psychology Unit, Goethe University Frankfurt, Frankfurt, Germany; University of Tartu, ESTONIA

## Abstract

Enjoyment is recognized as a determinant of physical activity habits. The enjoyment of engaging in physical activity can be measured with the Physical Activity Enjoyment Scale (PACES). Later versions of this scale have been shortened to capture the component of subjective feeling, which has been validated using German-speaking samples. The aim of this study was to examine internal consistency, factorial validity, criterion-related validity, test-retest reliability, and measurement invariance (across gender and languages) in an English-speaking population. Data on physical activity enjoyment and self-reported physical activity were collected through an online survey with a test-retest design (n = 276,189 female, M = 42.55, SD = 16.81 years) conducted from September 2023 to December 2023. In addition, a German-speaking sample (n = 1017, 497 female, M = 29.77, SD = 13.54 years) was analyzed to assess measurement invariance with respect to language. McDonald’s omega at time 1 was ω = 0.95. The confirmatory factor analysis supports the assumption of the unidimensional structure of PACES-S (χ^2^ = 19.8, df = 2, p < 0.05; CFI = 0.984; RMSEA = 0.180, 90% CI [0.113–0.256]), as the model fit can be estimated as good in view of the CFI value, while the sensitivity of the χ^2^ test is very high and the RMSEA may underestimate the model fit. Although the RMSEA value is higher than the conventional threshold, the small number of degrees of freedom may have a significant impact on this indicator. The criterion-related validity for light physical activity was r(107) = 0.26 (p *<* 0.05). The retest reliability was r(199) = 0.69 (p < 0.05). Further, the results supported measurement invariance across gender and partial measurement invariance across languages. Overall, the English PACES-S demonstrated good psychometric properties specifically for light intensity of physical activity, and can serve as an economical instrument to assess physical activity enjoyment.

## Introduction

Promoting the repetition of physical activity behavior is a central concern within the field of exercise psychology, as it is essential to continuously engage in physical activity to reap the associated health benefits [1,2]. This necessity is underscored by the fact that increased levels of overall physical activity and reduced sedentary time were associated with a decrease in the risk of premature mortality among middle-aged and older adults [3]. However, dropout in physical activity programs continues to remain as a significant challenge despite these distal rewarding consequences [4]. This highlights the importance of immediate advantages, such as perceived enjoyment, in maintaining or even making physical activity habitual [5–7]. The association between physical activity enjoyment and physical activity was robustly demonstrated in a meta-analysis, showing that higher levels of enjoyment are linked to greater adherence to physical activity routines [8].

In terms of measuring affective determinants, it is imperative to distinguish between automatic affective responses and conscious emotions [9]. Affective responses stemming from automatic processes are elicited from a stimulus that bypasses cognitive reflection. This automatic affective response can be differentiated by its valence ranging from positive to negative [10]. In contrast, emotions are manifested in the conscious processes based on cognitive appraisal of an observed stimulus and are thus comparatively slower to develop [9]. Their greater complexity compared to automatic affective responses is also evident in theories that posit emotions to be composed of various components, namely cognitive appraisals, neurophysiological processes, motivational tendencies, motor expressions, and subjective feelings [11].

The unique methods on how affective determinants are formulated in each processes also necessitates distinct approaches to measure them [12]. For instance, affective responses in the automatic processes can be measured by administering the Feeling Scale (asking “How do you feel right now?”), which is an established instrument that can be employed multiple times during an exercise session [13]. This approach makes it possible to depict the valence of the affective response when exercising. From individual patterns of the Feeling Scale values, conclusions can then be drawn regarding which intervals of the exercise induced the experience of pleasure or displeasure [14]. In contrast, measuring cognitively appraised emotions can be conducted by administering a scale post-exercise/physical activity session, such as the “Physical Activity Enjoyment Scale” (PACES; “Please rate how you feel at the moment about the physical activity you have been doing”), which was developed and validated across two studies [15].

Furthermore, it is important to consider not only acute affective or emotional experiences (i.e., automatic affective responses and conscious emotions), but also how they manifest in cognitive processing such as reflection or learning [9,16]. PACES is also appropriate for the use in studies that seek to measure a general cognitive reflection on the overall enjoyment expected from engaging in physical activity based on past emotional experiences [8]. For example, the item stem “When I am active” can be used in large-scale, representative studies where the focus is not on the acute emotion immediately after physical activity, but on how the participants feel during physical activity in general [17].

PACES is therefore widely used for measuring physical activity enjoyment [18]. Though overtime, researchers have noted some limitations in the scale (for an overview see also [19]). For example, validation studies revealed methodological problems of the common variance of negatively and positively formulated items [20–22]. Interestingly the scale’s original creators have raised the question of whether enjoyment should be viewed as a unidimensional construct [15], which lead to excluding items that were associated with preconditions or consequences of enjoyment (e.g., “it makes me depressed”), rather than the experience of enjoyment itself (e.g., “I enjoy it”) [23]. Another study which recruited older adults to reflect age-related changes in emotion regulation and emotion judgement was conducted to further refine the scale [24]. Here, a panel of experts was instructed to select only items related to psychological and social well-being. The resulting 8-item version was subjected to a factor analysis in another study, with the two factors that produced a better model fit than a unidimensional model being labeled “fun” (i.e., pleasurable entertainment) and “satisfaction” (i.e., momentary experience) by the authors [25].

PACES-S takes the next step towards scale refinement and efficiency [19]. It measures the cognitive reflection of the conscious emotion physical activity enjoyment with the associated subjective feeling considered its central emotional component [9,11,19]. While PACES-S was initially validated using data from youth participants [19], a subsequent validation study also demonstrated good reliability and validity of the instrument for an adult population [26]. Further, previous tests support PACES-S to be used invariantly across genders [26].

Overall, PACES-S presents convincing strengths, including lower participant item burden (4 item scale), and validity across the age range and between genders. However, the two validation studies were limited to German-speaking populations [19,26]. Given its potential for use in large-scale cross-cultural studies, it is important to investigate whether the underlying factorial structure, factor loadings, measurement intercepts, and residuals are consistent across languages to rule out bias due to measurement error [27].

The purpose of this study was to validate an English version of PACES-S. The primary objective of this study was to test psychometric properties which included the internal consistency, factorial validity, criterion-related validity, test-retest reliability, and gender invariance of PACES-S in an English-speaking population. The secondary objective was to assess whether the measure is invariant across English- and German-speaking populations.

## Methods

### Procedure

The psychometric properties of PACES-S within an English-speaking sample (Sample 1) were investigated using a test-retest design, gathering self-report data through online questionnaires. The two measurement points were separated by a four-week interval. Physical activity enjoyment and sociodemographic information were evaluated during the initial measurement, while the second measurement focused on assessing physical activity enjoyment and self-reported physical activity. Data collection was conducted from September 20, 2023, to December 30, 2023. Ethical clearance was secured from the Indiana University Institutional Review Board. Informed consent was obtained from all participants prior to their involvement in the study. All consented subjects provided a signature as documentation of consent.

To conduct an invariance analysis across languages, we additionally examined self-report data on physical activity enjoyment obtained from a German cross-sectional study, utilizing paper-and-pencil questionnaires (Sample 2). This particular sample was previously incorporated in the aforementioned investigation of the psychometric properties of PACES-S within an adult German population [26]. Data collection was conducted from April 2019, to December 2019. Ethical approval was secured from the ethics committee at Humboldt University Berlin. All participants provided written informed consent prior to their involvement in the study.

### Sample 1

English-speaking participants were recruited through Prime Panels provided by CloudResearch, a national online platform that connects researchers with over 50 million potential participants across the United States. CloudResearch’s sampling methods have been shown to yield participants that are representative of the broader U.S. population [28]. Potential participants can utilize a search function on the website to browse studies currently recruiting participants. Each study is displayed under tabs that include the study title and relevant keywords. By selecting a study link, individuals are directed to the survey’s initial page, where the consent form is provided. Participants can then give their digital consent and proceed with the survey if they choose to participate. Each respondent received a $5.00 compensation for their participation. Inclusion in the study required only two criteria: (a) being a minimum of 18 years old and (b) being fluent in the English language. The study comprised a total of 276 participants (189 female, 86 male, 1 with missing data) with an average age of 42.55 years (SD = 16.81, ranging from 18 to 85 years). From those participants, 202 (137 female, 64 male, 1 with missing data) individuals with a mean age of 46.25 years (SD = 17.81; range = 20 to 85 years) took part in the second measurement for the test-retest reliability.

### Sample 2

German-speaking participants were recruited through personal contact from university courses, fitness gyms, or sports clubs. These participants attended recreational and amateur sports and exercise programs at various university sports facilities, various German sports clubs, or commercial providers. To be included in the study, only two criteria needed to be met: (a) being a minimum of 18 years old and (b) being fluent in the German language. The study comprised a total of 1017 participants (497 female, 2 with missing data) with an average age of 29.77 years (SD = 13.54, ranging from 18 to 83 years). Of these participants, 394 took part in individual sports and exercise programs, and 621 in group or team sports and exercise programs (2 with missing data).

### Measures

#### PACES-S

Physical activity enjoyment was measured in both samples. Regarding Sample 1, we used a English version of the PACES short version (PACES-S, [19]), with the item stem “When I am physically active“, that were answered with the four items “I like it”, “I find it pleasurable”, “It is very pleasant”, and “It feels good” on a five-point Likert scale ranging from (1) strongly disagree to (5) strongly agree. In German (Sample 2), the item stem was “Mich zu bewegen” (literally translated “being physically active”). Overall enjoyment was calculated as the mean of the four items, with higher values indicating higher levels of enjoyment. Two prior investigations have affirmed the psychometric properties of the German PACES-S in both a youth [19] and an adult population [26]. Regarding reliability, the latter evaluated McDonald’s omega in three studies and concluded that the level of internal consistency was acceptable to good (values between 0.78 and 0.88). Further, it demonstrated moderate test-retest reliability (r = 0.73). Concerning factorial validity, the three studies supported the unidimensional structure of the instrument using confirmatory factor analysis (e.g. χ^2^ = 10.0; df = 2; p < 0.01; CFI = 0.992; RMSEA = 0.063, latent factor loadings between 0.63 and 0.75). In terms of criterion-related validity, physical activity enjoyment was significantly positively correlated with self-reported physical activity (r = 0.40). Moreover, the investigation showed the measurement’s invariance across gender.

#### International physical activity questionnaire

In Sample 1, self-reported physical activity was assessed based on relevant items from the modified International Physical Activity Questionnaire (IPAQ, [29]), which referred to the last two weeks. Participants were asked about the number of days per week and the average number of minutes per session they engaged in light (defined as any physical activity that does not cause a noticeable change in breathing) and moderate (defined as any physical activity that is not exhausting or only makes one breathe somewhat harder than normal) physical activity. The product of the days per week and the amount of minutes per session yielded the average minutes per week that participants were physically active in the two different categories of physical activity.

### Statistical analysis

The present paper contains data from two samples. First, we examined psychometric properties (i.e., internal consistency, factorial validity, criterion-related validity, test-retest reliability, and gender invariance) of PACES-S in an English-speaking sample. Second, we additionally utilized a dataset from a German-speaking sample to conduct invariance analysis across languages.

As a first step, Little’s MCAR test [30] was performed to check patterns of missing values. In addition, all physical activity information (assessed with the IPAQ) was checked for plausibility. If days per week exceeded 7, the value was corrected to 7. Questionable values for the amount of minutes per session (e.g., 2 minutes of light physical activity per session) were neither corrected nor deleted. Correlations were calculated once with and once without the implausible values or once with and once without the corrected values in order to check the robustness of the results. Further, the descriptive statistics mean (M) and standard deviation (SD) were calculated and the differences in the means between the two samples were tested for significance using the Welch-test (see also [31]). The threshold for significance was .05 for all analyses.

#### Internal consistency

To evaluate internal consistency, we determined McDonald’s omega using the SPSS macro developed by Hayes and Coutts [32].

#### Factorial validity

In order to assess the unidimensional factor structure identified in previous studies of the German short version [19,26], a confirmatory factor analysis (CFA) was performed using full-information maximum likelihood estimation in IBM SPSS AMOS 28 [33]. This estimation method was chosen as it allows for an unbiased assessment of missing data [34]. Overall model fit was evaluated using the χ^2^-statistic. Here, a non-significant p-value indicates a good fit for the model [35]. However, for large samples, the test is highly sensitive, detecting even minor deviations between the observed and model-implied covariance matrices [36,37]. As a result, it can reject the null hypothesis of a good model fit even when the model’s inaccuracies are negligible. Therefore, we additionally used the comparative fit index (CFI), which assesses the relative improvement in fit by comparing the proposed model to a baseline model, and values in the range of 0.90 to 0.95 are considered indicative of an acceptable model fit, while values exceeding 0.95 suggest a good model fit [37]. Further, we applied the root mean square error of approximation (RMSEA) as an indicator of how closely the model fits the data, with values below 0.05 indicating of a good model fit and values between 0.05 and 0.08 indicating an acceptable model fit [38]. Additionally, for a good model fit, the 90% confidence interval around the RMSEA point estimate should encompass zero [39].

#### Criterion-related validity

To assess criterion-related validity, the respective correlations between physical activity enjoyment measured with PACES-S and the two categories of physical activity (i.e., light and moderate physical activity) were calculated.

#### Test-retest reliability

To assess test-retest reliability, we determined the Pearson product-moment correlation between PACES-S scores obtained during the first and second measurements.

#### Measurement invariance for gender

A multi-group confirmatory factor analysis (CFA) was conducted by testing four nested models using IBM SPSS AMOS 28 [33] to examine the measurement invariance for gender (male vs. female) [27]. As such, certain parameters (e.g., factor-loading regression paths) are systematically examined in a reasonably ordered and progressively restrictive manner, starting with an unconstrained model to test the validity of factorial structure, also known as configural invariance (Model A) [40,41]. In the following models, cross-group equality constraints were imposed as follows [27]: In Model B, the factor loadings were constrained equal across groups. In Model C, the constraints of the previous model were retained and additionally, the measurement intercepts were constrained to equality. Finally, in Model D, in addition to factor loadings and measurement intercepts, also the residuals (i.e., regression residual variances for all items) were constrained equal [27]. As such, Model B tested metric invariance, Model C tested scalar invariance, and Model D tested residual invariance [41].

Model fit parameters of all models were simultaneously estimated for both groups [40]. This involved the consideration of χ^2^ difference tests and change in Comparative Fit Index-values (ΔCFI) as an alternative criterion for evaluating measurement invariance [42]. If the χ^2^ for model comparison is not statistically significant or ΔCFI-values are ≤ .01, the hypothesis of invariance is maintained. However, if a model had to be rejected according to these criteria, partial invariance models (e.g., partial metric invariance model) were tested. These models allow certain parameters (e.g., factor loadings) to differ between groups and this freely estimated subset of parameters may reveal non-invariant items [40,41,43].

#### Measurement invariance across languages

The same procedure as described above for multi-group CFA was applied to examine the two samples, German-speaking and English-speaking participants, in order to assess measurement invariance across languages.

## Results

### Descriptive statistics

In Sample 1, the percentage of missing values was 0.4% for items 1 and 2, 1.8% for item 3 and 1.4% for item 4 in the first measurement. In sum, for the four items, 11 values (0.996%) distributed among 5 subjects were missing. The Little’s MCAR test was significant (χ^2^ = 43.2, df = 5, p < 0.05), indicating that missingness in the data was related to the data. Applying listwise deletion, we removed the participant who had no values for PACES-S and imputed values for the remaining four participants with missing values using the expectation-maximization algorithm (EM algorithm; [34,44]).

In the second measurement, the percentage of missing values was 0% for items 1 and 2 and 0.5% for items 3 and 4. In sum, for two of the four items, 2 values (0.249%) distributed among 2 subjects were missing. The Little’s MCAR test was not significant (χ^2^ = 7.6, df = 6, p = 0.27), indicating that missingness in the data was not related to the data. We imputed values for the two participants with missing values using the expectation-maximization algorithm again (EM algorithm; [34,44]).

Regarding Sample 2, the percentage of missing values was 0% for items 1 and 4 and 0.1% for items 2 and 3. In sum, for two of the four items, 2 values (0.049%) distributed among 2 subjects were missing. The Little’s MCAR test was not significant (χ^2^ = 12.1, df = 6, p = 0.06), indicating that missingness in the data was not related to the data. Again, we imputed values for the two participants with missing values using the expectation-maximization algorithm (EM algorithm; [34,44]).

Means and standard deviations of the individual PACES-S items and the overall scale in Sample 1 (for both measurement times) and Sample 2 are shown in Table 1. There was a significant difference (t(309.3) = 11.31, p < 0.05) between PACES-S values of Sample 1 in the first measurement (English) and Sample 2 (German), with mean PACES-S values 0.78 points (95%-CI [0.64, 0.91]) lower in Sample 1. There was also a significant difference (t(215.8) = 9.09, p < 0.05) between PACES-S values of Sample 1 in the second measurement (English) and Sample 2 (German), with mean PACES-S values 0.79 points (95%-CI [0.62, 0.96]) lower in Sample 1.

**Table 1:** Descriptive statistics.

	Sample 1				Sample 2	
	M_1_	SD_1_	M_2_	SD_2_	M	SD
When I am physically active I like it.	3.82	1.19	3.97	1.28	4.71	0.55
When I am physically active I find it pleasurable.	3.65	1.19	3.56	1.31	4.37	0.75
When I am physically active it is very pleasant.	3.49	1.19	3.34	1.35	4.20	0.83
When I am physically active it feels good.	3.77	1.19	3.84	1.29	4.57	0.61
PACES-S	3.69	1.11	3.68	1.20	4.46	0.53

M1 = mean at first measurement; SD1 = standard deviation at first measurement; M2 = mean at second measurement; SD2 = standard deviation at second measurement.

Regarding self-reported physical activity, 13 individuals reported more than 7 days per week and were corrected to 7. Six individuals reported questionable values for minutes per session (< 10 minutes), but these values were retained. For light physical activity, the mean was M = 158.42 minutes per week (SD = 222.77) and for moderate physical activity, the mean was M = 110.71 minutes per week (SD = 104.79).

### Psychometric properties

#### Internal consistency

Concerning the reliability, the values for McDonald’s omega (ω = 0.95 at the first measurement; ω = 0.94 at the second measurement) indicated an excellent internal consistency of PACES-S [45].

#### Factorial validity

Regarding the factorial validity of PACES-S, the model showed a good fit to the data based on the CFI value, which was close to 1 (χ^2^ = 19.8, df = 2, p < 0.05; CFI = 0.984; RMSEA = 0.180, 90% CI [0.113–0.256]). However, the χ^2^-test was significant and the 90% confidence interval for RMSEA did not cover 0, but given the high sensitivity of the χ^2^-test [37] and the low degrees of freedom, this does not necessarily imply that the model would not fit [46].

The standardized regression weights (i.e., factor loadings; see the left column of Table 2) correspond to the correlation between the latent factor and each manifest indicator variables, while the squared standardized factor loadings (see the right column of Table 2) correspond to the proportion of the variance in the indicator variables explained by the latent factor [47]. All individual items significantly loaded on the latent factor with an explained variance between R^2^ = 0.781 and R^2^ = 0.882.

**Table 2:** Standardized regression weights and explained variance.

	Factor loadings	R^2^
Item 1	0.884	0.781
Item 2	0.939	0.882
Item 3	0.904	0.818
Item 4	0.894	0.799

R^2^ = squared factor loadings; Factor loadings = standardized regression weights.

#### Criterion-related validity

In terms of the criterion-related validity, the correlation between light physical activity and PACES-S [r(107) = 0.26, p < 0.05] but not between moderate physical activity and PACES-S [r(96) = 0.01, p = 0.91] was significant. These results were the same when the individuals with the corrected values (when days per week > 7 days) or implausible values (minutes per session < 10 minutes) were excluded.

#### Test-retest reliability

Regarding test-retest reliability, the correlation between PACES-S scores obtained during the first and second measurement was significant [r(199) = 0.69, p < 0.05].

#### Measurement invariance for gender

Regarding gender invariance, as shown in Table 3, the χ^2^-difference test was not significant for any model (Models B to D). Further, because the difference in CFI did not exceed 0.01 for any comparison, the results suggest that the instrument is invariant across gender [42]. Table 3 also shows that RMSEA was not always within the recommended ranges [38], but given the small sample size and low degrees of freedom, this does not necessarily imply that the model would not fit [46].

**Table 3:** Analysis of gender invariance across different models.

Model	χ^2^	df	p	CFI	RMSEA	ΔCFI	Δχ^2^	Δdf	p
Model A	26.6	4	< 0.05	0.979	0.114				
Model B	28.7	7	< 0.05	0.979	0.107	0.000	2.1	3	.55
Model C	30.5	10	< 0.05	0.981	0.087	0.002	1.8	3	.61
Model D	36.7	14	< 0.05	0.979	0.077	0.002	6.2	4	.18

χ^2^ = chi-square; df = degrees of freedom; p = probability value; CFI = Comparative Fit Index; RMESA = Root Mean Square of Approximation; ΔCFI = difference in CFI; Δχ^2^ = difference in chi-square; Δdf = difference in degrees of freedom.

#### Measurement invariance across languages

Regarding language invariance, the first row of Table 4 shows that Model A, which tested configural invariance, had a good model fit based on the CFI value and an acceptable model fit based on the RMSEA value, which is an indication of the equivalence of model form [41]. However, as shown in the second row, the difference in CFI for the comparison between Model A and B_1_ exceeded 0.01, and the χ^2^-difference test was significant, indicating metric noninvariance. As a way of dealing with metric noninvariance, factor loading constraints were relaxed until partial metric invariance was found [41]. Following a forward approach, we first removed all factor loading constraints except for item 2, i.e., the item with the first factor loading to be estimated [40,41]. The model fit still indicated noninvariance (model not shown in Table 4). Therefore, the factor loading of item 2 was freely estimated rather than constrained to be equal in the next model and only the factor loading of item 3 was constrained to be equal, but the model fit still indicated noninvariance. Thus, the factor loadings of items 2 and 3 were freely estimated rather than constrained to be equal and only the factor loading of item 4 was constrained to be equal in the next model, Model B_2_ (see Table 4). The difference in CFI for the comparison between Model A and B_2_ did not exceed 0.01, and the χ^2^-difference test was not significant, indicating partial metric invariance with the factor loading parameter of item 4 being equal across groups [40]. In sum, we concluded that items 2 and 3 were noninvariant, but that item 4 was invariant [40]. As such, only the equality constraints for item 4 were maintained when moving on to Model C. Subsequently, scalar invariance (Model C) and residual invariance (Model D) were found. Regarding the comparisons between Model B_2_ and C as well as Model C and D, while the χ^2^-difference tests were significant, the differences in CFI did not or only slightly exceed 0.01, supporting invariance [42]. In sum, the results suggest that the instrument is partially invariant across languages.

**Table 4:** Analysis of invariance across languages.

Model	χ^2^	df	p	CFI	RMSEA	ΔCFI	Δ χ^2^	Δdf	p
Model A	29.8	4	< .05	0.988	0.071				
Model B_1_	89.7	7	< .05	0.962	0.096	0.026	59.9	3	< .05
Model B_2_	32.0	5	< .05	0.987	0.065	0.001	2.2	1	0.14
Model C	36.9	6	< .05	0.986	0.063	0.001	4.9	1	< .05
Model D	61.8	8	< .05	0.975	0.072	0.011	24.9	2	< .05

χ^2^ = chi-square; df = degrees of freedom; p = probability value; CFI = Comparative Fit Index; RMESA = Root Mean Square of Approximation; ΔCFI = difference in CFI; Δχ^2^ = difference in chi-square; Δdf = difference in degrees of freedom.

## Discussion

The purpose of this study was to test the psychometric properties of PACES-S [19,26] in an English-speaking population and to examine its measurement invariance compared to a German-speaking sample. When employing PACES-S in diverse settings, it is important to test the measurement’s invariance across various population attributes. Overall, the psychometric tests met reliability and validity criteria. In terms of reliability, the results indicate excellent internal consistency and acceptable test-retest reliability. Regarding factorial validity, the results point to a good model fit based on the CFI value. Additionally, the results for criterion-related validity indicate a significant positive relationship with light, but not moderate physical activity. With regard to the measurement invariance, the instrument appears to be invariant across gender, but invariance across languages was only partially evident. Overall, these findings improve the understanding of how to measure physical activity enjoyment with a practical and cost-effective tool. The English version of PACES-S has the potential to be utilized in multicultural contexts, such as global, large-scale physical activity surveys or intervention studies.

The reliability values obtained in this study, namely an McDonald’s omega exceeding 0.90 at both measurement points, were higher than the values found in a validation study of PACES-S in German-speaking adults (between 0.78 and 0.88) [26]. The authors attributed the acceptable but not excellent values to the possibility of altered variance due to a restriction of range, given the recruitment of two of their samples in sports contexts [48]. Notably, the English-speaking sample used in the present study included not only individuals engaged in exercise contexts, and the mean scores of PACES-S were significantly lower than those of the German-speaking sample, which had also been used in the previous validation study. In sum, our values were more comparable to the excellent values for internal consistency of the long version of PACES [15]. Further, the test-retest reliability (r = 0.69) was slightly below the recommended value of 0.70 [45], but still comparable to the results of the study on PACES-S in German adults [26] and to a study on the long version [20].

Regarding factorial validity, a good model fit was revealed based on the CFI value [37], while the χ^2^-test was significant and the RMSEA value was above 0.08. However, the sensitivity of the χ^2^-test is very high [37] and the RMSEA can also underestimate the model fit when the degrees of freedom is low [46], which was the case in the present study. In general, our results tend to support the assumption of the unidimensional structure of PACES-S, as also emphasized in the study on German adults, while the factor loadings and the explained variance were even higher than in this previous study [26]. The question of whether enjoyment is a unidimensional construct or whether it can be divided into components was already raised in the study on the development of the original long-version PACES [15]. Certain items associated with preconditions or consequences of enjoyment were excluded from PACES in previous studies, focusing on the experience of enjoyment itself [23]. Following a similar approach, PACES-S was developed with the aim to only include those items that reflect the subjective experience of enjoyment as the most essential component of an emotion [11,19]. Our results tend to support that PACES-S consists of only one latent factor that could represent the cognitive reflection about the overall subjective experience of physical activity enjoyment [9].

In terms of the criterion-related validity, the results indicate that PACES-S is positively correlated with light, but not moderate physical activity. In contrast, previous studies have found an association between moderate to vigorous physical activity and physical activity enjoyment when using PACES-S [26] and the long version [22] in German adults. It is important to note differences in the samples that may explain these findings. For example, while the average number of minutes of moderate to vigorous physical activity per week in the previous validation study of PACES-S in German adults was about 200 minutes, in contrast, the average for moderate physical activity in the present study was only about 111 minutes per week. This could be explained by the fact that the mean age in the previous study was 22.30 (SD = 3.25, range = 18 to 31) and in the present study 42.55 years (SD = 16.81, range = 18 to 85). As such, the lack of a significant correlation with moderate physical activity might be due to socio-cultural differences or the average age of the sample, which could influence the type of activity performed.

Tests for measurement invariance found PACES-S to be invariant across gender, which aligns with findings in the previous validation study with German-speaking participants [26].

Finally, the measurement invariance analyses showed that invariance across languages appears to be partial. In a first step, this study tended to demonstrate configural invariance, meaning that the basic model structure (4 items loading on a latent factor) of the construct was acceptable for both groups [41]. In the second step, metric invariance was examined. Metric invariance would mean that the contributions of each item (i.e., factor loading) to the latent factor (i.e., the construct of physical activity enjoyment) are similar across the two groups [41]. However, the model fit of the metric invariance model was significantly worse than that of the configural invariance model. This finding of metric noninvariance suggests that at least one factor loading was not equivalent across the two groups [41] and that there may be disagreement between the two groups about the manifestation of the construct [42]. How much the frequently occurring finding of metric noninvariance affects group comparisons depends on the number of invariant items–with a small number of invariant items no problematic bias is to be expected [42,43]. Therefore, it is recommended to relax the constraints of overall construct-level metric invariance (i.e., equality of *all* factor loadings) at the item level (i.e., identification of metric noninvariant items to detect partial metric invariance) [42,43]. In the present study, there was evidence that item 2 “When I am physically active I find it pleasurable” (German: “Mich zu bewegen genieße ich”) and item 3 “When I am physically active it is very pleasant” (German: “Mich zu bewegen ist sehr angenehm”) may be the metric noninvariant items. These items may contribute more to the construct of physical activity enjoyment in the English-speaking than in the German-speaking sample. This may be due to the linguistic proximity between the words of the items. It could be that the words “pleasurable” and “pleasant” are linguistically closer in English and are seen more as representatives of the category “enjoyment”, which is in fact defined as an emotion associated with feelings of “pleasure” [19]. In contrast, in German, the words “genießen” and “angenehm sein” may be linguistically further apart and belong to different linguistic categories than “Freude”. Cultural differences in how these concepts are perceived may also play a role. In English-speaking cultures, “pleasurable” and “pleasant” may be more strongly and intuitively associated with the construct of physical activity enjoyment than the German equivalents “genießen” and “angenehm sein”. In German-speaking cultures, these terms may not be the first words associated with physical activity or may not be as closely associated with the immediate, sensory pleasure that “pleasurable” and “pleasant” imply. In particular, “angenehm sein” (implying mild satisfaction) may not capture the active nature of physical activity enjoyment as effectively. However, this can only be speculated here and would be the subject of future research, preferably from the qualitative research stream.

In the third and fourth step, both scalar and residual invariance tended to be supported for the metric invariant items 1 and 4. This means that the mean differences in physical activity enjoyment capture all mean differences in the common variance of the items and that the sum of the variance of the items that is not shared with the latent factor and the error variance is comparable in both groups [41]. Therefore, PACES-S fulfills the conditions for comparing latent means [42].

### Strengths and limitations

The validation of an English version of PACES-S presents several strengths worth noting. In addition to other psychometric properties, measurement invariance across languages was examined using structural equation modeling. Specifically, configural, metric, and scalar invariance were tested as prerequisites for comparing latent means, as well as residual invariance, which was often omitted in other studies [41]. By considering partial invariance models, metrically noninvariant items were uncovered. While the present study remains speculative in the interpretation of this finding, future studies could explore the linguistic comparability of PACES-S in greater depth, both at the content level using qualitative studies and at the formal level using artificial intelligence. Regarding the latter, the use of the web program “Survey Quality Predictor 3.0” [see also 49] seems promising. This tool could be used to reveal formal differences resulting from translations, to suggest corrections and finally to estimate the quality of items, so that it could contribute to greater comparability of items across languages.

A limitation of this study was that the scale demonstrated stronger psychometric properties towards lower intensities of physical activity. Additional tests are warranted to confirm if the measure would demonstrate predictive validity for higher intensities of physical activity. In this context, it should be noted that the scale could also be used and validated in competitive sports. For example, research has demonstrated the significant role of emotional regulation, particularly in high-pressure situations, on the enjoyment and performance in precision sports such as archery [50].

Another limitation of the present study is the smaller sample size of the English-speaking sample compared to the German-speaking sample, which may have limited the statistical power in the criterion-related validity analyses. However, given the greater variance in terms of physical activity enjoyment, the English-speaking sample seems to be more representative than the larger German-speaking sample recruited from a sports context. A possible limitation, given that the German sample was recruited in sports contexts, could be the altered variance due to range restriction. Future studies could consider matching samples on variables such as age or amount of physical activity to make them more comparable, differing only in terms of language. In this regard, it would also be advisable to translate the scale into additional languages and to examine measurement invariance across different languages (see also [51]). Further, the use of self-report data for physical activity to test the criterion-related validity constitutes a limitation of the present study, given the discrepancy between self-reported and device-based measures of physical activity [52]. Self-reported measures may be subject to recall bias or social desirability bias.

## Conclusions

In conclusion, the results of the present study supported psychometric properties of PACES-S, including excellent internal consistency and acceptable test-retest reliability, as well as factorial validity, criterion-related validity regarding light physical activity, and measurement invariance across gender. Partial measurement invariance across languages was also demonstrated. The items “I find it pleasurable” and “it is very pleasant” may be metric non-invariant items and should therefore be further analyzed in future qualitative studies for their linguistic accuracy in relation to physical activity enjoyment in German and English, and possibly other languages. In sum, this scale serves an English version of the previously validated German language PACES-S measure [19,26]. These scales can assist in conducting cross-group comparative research with minimized measurement bias. In addition, this more economical version of PACES, consisting of only 4 items, is an instrument suitable for large-scale or ambulatory assessment studies. Further research is encouraged to assess if the scale can predict higher intensities of physical activity, and would exhibit residual invariance across languages.
